# Is living near green areas beneficial to mental health? Results of the Pró-Saúde Study

**DOI:** 10.11606/s1518-8787.2019053001008

**Published:** 2019-09-12

**Authors:** Patricia Amado Barreto, Claudia Souza Lopes, Ismael Henrique da Silveira, Eduardo Faerstein, Washington Leite Junger

**Affiliations:** I Universidade do Estado do Rio de Janeiro. Instituto de Medicina Social. Programa de Pós-Graduação em Saúde Coletiva. Rio de Janeiro, RJ, Brasil; II Universidade do Estado do Rio de Janeiro. Instituto de Medicina Social. Departamento de Epidemiologia. Rio de Janeiro, RJ, Brasil

**Keywords:** Adult, Mental Disorders, prevention & control, Green Areas, Socioeconomic Factors, Mental Health, Cross-Sectional Studies

## Abstract

**OBJECTIVE:**

To investigate the association between exposure to green areas in the surroundings of the residence and the presence of common mental disorders among adults, according to different income strata.

**METHODS:**

Cross-sectional study with 2,584 participants from the Pró-Saúde Study (2006), residing in the city of Rio de Janeiro. Common Mental Disorders were measured using the General Health Questionnaire (GHQ-12) and exposure to green areas was measured using the normalized difference vegetation index, in buffers with radiuses between 100 and 1,500 meters around the residence. We used the mean and maximum normalized difference vegetation index categorized into quartiles. The study population was divided into three subgroups, according to the income: low, intermediate, and high. Odds ratios and their 95% confidence intervals were estimated with logistic regression models. The models were adjusted by sex and age, with and without inclusion of physical activity practice.

**RESULTS:**

The proportion of common mental disorders was 30% and 39% among men and women, respectively. The results of the adjusted models showed an inverse association between the presence of green areas in the surroundings of the residence and the occurrence of common mental disorders, in the buffer of 200 meters in the intermediate-income group and in the buffers of 400 and 1,500 meters in the low-income group. The odds ratio ranged from 0.52 (buffer of 1,500 meters) to 0.68 (buffer of 200 meters). The association found was independent of physical activity practice.

**CONCLUSIONS:**

The evidence found suggests the existence of a beneficial effect of urban green areas on the mental health of lower-income individuals. These findings can help in understanding how the urban environment can affect the mental health of the population.

## INTRODUCTION

The physical environment of the neighborhood, including aspects such as neighborhood landscape, agglomeration and noise level, school and health establishments, recreational public facilities and public transportation, contributes to the quality of life and the feeling of satisfaction of residents in relation to the neighborhood, house, and community^1,2^. Life satisfaction is related to mental disorders^3^ and the physical characteristics of the neighborhood environment may have the potential to influence mental health by the improvement of the quality of life of its residents. Studies show that the availability of green areas (squares, parks, gardens, woods, and wooded streets) has the potential to promote the sensation of relaxation, well-being, and social cohesion^4,5^, in addition to promoting the practice of physical activities^6,7^, which contributes to the promotion of mental health in the population.

Studies have shown a high prevalence of common mental disorders (CMD) in urban areas in the country^8,9^. These disorders are characterized by the presence of symptoms of anxiety and depression that, although insufficient for the characterization of a psychiatric diagnosis, can cause substantial suffering and impairment in the lives of individuals^10^.

Evidence shows that people with lower socioeconomic status are more dependent on the surrounding area and present worse health conditions in a general way^11^. Measures aimed at promoting the improvement of physical characteristics in urban centers could impact the mental health of the most deprived portion of the Brazilian population, helping to reduce inequality in relation to quality of life.

Most studies on this topic are concentrated in high-income countries, and the association between exposure to green areas and mental health is still inconsistent, with scarce evidence on the subject in low- and middle-income countries, such as Brazil and other countries in Latin America^4,12^. The mediation of potential beneficial effects by favoring physical activity practices still presents no conclusive results^6,13^. Rio de Janeiro, the second largest city in the country, has a natural environment that, albeit exuberant, is unevenly distributed throughout the city^14^. Considering this context, this study aimed to investigate the association between the presence of green areas in the surroundings of the residence and the occurrence of CMD among adult residents and between different economic strata.

## METHODS

### Study Design and Population

This is a transversal study inserted in a longitudinal study, the Pró-Saúde Study (EPS). The EPS aimed to investigate the role of health and morbidity determinants among administrative technical staff of a university located in the city of Rio de Janeiro, Brazil^15^.

For this study, we used data from the third phase of the EPS, performed in 2006. Of the 3,604 participants of this phase, only those who resided in the city of Rio de Janeiro and whose addresses were successfully georeferenced were included in the study, totaling 2,584 individuals.

### Individual Variables

Data was collected using a self-completed, multidimensional questionnaire, covering physical and mental morbidity, access to and use of health services, and socioeconomic aspects, among others. We used procedures aimed at quality of information, such as pilot study, test-retest to ensure instrument reliability, and procedures of independent double typing^15^.

The outcome analyzed in this study was the occurrence of common mental disorder, measured by the 12-item version of the General Health Questionnaire (GHQ-12), a screening tool for non-psychotic mental disorders, including anxiety, depression, and somatic symptoms. Each question has four answer options, two considered positive and two considered negative, which, respectively, add 1 or 0 to the GHQ score. The participant is classified as positive for CMD if the score is greater than or equal to 3^16^.

Other individual variables considered in the study were: sex, age in years (25–35, 36–45, 46–55, 56–65, 66–75), net monthly income (up to BRL 500, BRL 501–1,000, BRL 1,001–1,500, BRL 1,501–2,000, BRL 2,001–2,500, BRL 2,501–3,000, BRL 3,001–4,000, BRL 4,001–5,000, above 5,000), and practice of physical activities (yes or no). This practice was assessed using the question: “In the last two weeks, have you practiced any physical activity to improve your health, physical condition, or with aesthetic or leisure purpose?”.

### Indicators for Exposure to Green Areas

To obtain the variables for exposure in the surroundings of the residences, the participants’ addresses were georeferenced using the geo-referencing function (Google Geocoding API) of Google’s application programming interface (API). The API relates the informed addresses with the Google Maps database to obtain latitude and longitude coordinates. The procedure was performed in the R 3.1.1 program.

Green areas, in this work, refer to any vegetation surrounding the residence, such as trees planted in sidewalks, flowerbeds or squares, public or residential gardens, reserves, parks or recreation areas, provided they are covered by some kind of vegetation. The exposure was estimated by the normalized difference vegetation index (NDVI).

The NDVI is estimated using satellite remote sensing images and measures the vegetation density of a region. The vegetation produces a pattern of low reflectance of the red wavelength and high reflectance near the infrared. The index is determined by the difference of the two bands divided by the sum. Each pixel of the image receives a NDVI value that can range between 1 and -1, assuming in practice values between -0.1 and 0.7. Negative values indicate the presence of water, ice, and clouds; values between -0.1 and 0.1 indicate uncovered surfaces; and values around 0.6 indicate dense green vegetation^a^.

The images were obtained from the Landsat 4-5 TM collection, of the United States Geological Survey – USGS, which has a precision of 30 meters. For 2006 and for the study region, three images showing less than 10% of clouds were available, corresponding to the months of February, August, and December. The NDVI of the study period was estimated by the mean of these three months. The participants’ exposure was measured by the maximum and mean NDVI in buffers*,* circular regions centered in the participants’ residences, with radiuses of 100, 200, 300, 400, 500, 1,000, and 1,500 meters. Buffers of different sizes were used to analyze sensitivity, since there is no consensus in the literature about the appropriate size. The mean NDVI is estimated by the sum of the NDVI of each pixel divided by the number of pixels within the buffer. The maximum NDVI refers to the highest NDVI value in a pixel within the buffer.

### Data analysis

The participants were stratified into three groups of income (low income: up to BRL 1,500, intermediate income: BRL 1,501–2,500, high income: above BRL 2,501). The associations between the exposure to green areas and the presence of CMD were tested using bivariate and multivariate logistic regression models in these three groups. We also analyzed two multivariate models, one without adjusting by the practice of physical activities and another one adjusted by this variable, to investigate its influence on the model. The correlation and the potential collinearity between the explanatory variables was tested by the Spearman correlation coefficient and the variance inflation factor (VIF). The analyses were performed in the R 3.1.1 program.

The research was initially approved in the baseline of the longitudinal study in 1999 by the Research Ethics Committee of the Pedro Ernesto Hospital and subsequently by the Research Ethics Committee of the Institute of Social Medicine of the State University of Rio de Janeiro (CAAE Registration 0041.0.259.000-11). All participants signed an informed consent form.

## RESULTS

Table 1 shows the presence of CMD according to individual variables in the study population. CMDs were more frequent among women, participants aged 25–35 years and 66–75 years, participants with lower income, and those who practiced no physical activity in the last two weeks.

**Table 1:** Occurrence of CMD, according to individual variables, in participants of the Pró-Saúde Study, residing in the city of Rio de Janeiro, Brazil, 2006.

Variable	Participants		Occurrence of CMD	
	
	n	%	%	p*
Sex				
Male	1,062	41.10	30.23	< 0.01
Female	1,522	58.90	39.36	
Age group (years)				
25–35	184	7.12	44.02	0.02
36–45	936	36.22	34.83	
46–55	1,094	42.34	36.20	
56–65	319	12.35	29.78	
66–75	50	1.93	42.00	
Net income (reais)				
≤ 500	52	2.01	57.69	< 0.01
501–1,000	150	5.80	42.67	
1,001–1,500	301	11.65	49.17	
1,501–2,000	373	14.43	36.73	
2,001–2,500	321	12.42	36.14	
2,501–3,000	366	14.16	34.97	
3,001–4,000	345	13.35	31.01	
4,001–5,000	249	9.64	30.12	
> 5,000	416	16.10	26.20	
Physical Activity Practice				
No	1,453	56.23	42.60	< 0.01
Yes	1,128	43.65	26.60	

*Chi-square test

The mean estimated for the mean NDVI was higher according to the buffer size, but always presenting low values. The lowest value was found in the buffer of 100 meters (0.099) and the highest value was found in the buffer of 1,500 meters (0.154). The mean of the maximum NDVI also increased according to the buffer size; the lowest value was 0.233 in the buffer of 100 meters and the highest value was 0.611 in the buffer of 1,500 meters.

Table 2 presents the results of the gross and adjusted analyses of the association between the presence of CMD and the exposure to green areas measured by the maximum NDVI, stratified by income. Model 1 presents the gross analyses, Model 2 presents the analyses adjusted by sex and age, and Model 3 presents the adjustment by the individual variables of model 2 plus the practice of physical activity. This table presents only the results of the buffers that showed a statistically significant association at 5% level.

**Table 2:** Associations between the occurrence of common mental disorders (CMD) and the exposure to green areas, measured by the maximum NDVI in buffers of different sizes, stratified by income. Pró-Saúde study, Rio de Janeiro, Brazil, 2006.

NDVI in the buffers (meters)	Model 1^a^	Model 2^b^	Model 3^c^	n
Low-income group (up to R$ 1,500.00)				503
Max NDVI (200)				
Q1 (0.034–0.217)	1.00	1.00	1.00	114
Q2 (0.218–0.314)	0.87 (0.53–1.42)	0.88 (0.53–1.46)	0.88 (0.53–1.48)	142
Q3 (0.315–0.427)	1.07 (0.64–1.77)	1.071 (0.637–1.80)	1.02 (0.60–1.72)	126
Q4 (0.428–0.699)	0.82 (0.49–1.37)	0.74 (0.43–1.26)	0.72 (0.42–1.24)	120
Max NDVI (400)				
Q1 (0.124–0.361)	1.00	1.00	1.00	144
Q2 (0.362–0.457)	0.76 (0.48–1.22)	0.81 (0.50–1.31)	0.78 (0.48–1.27)	137
Q3 (0.458–0.535)	0.97 (0.57–1.57)	1.01 (0.60–1.70)	0.99 (0.58–1.67)	105
Q4 (0.536–0.714)	0.63 (0.39–1.04)	0.62 (0.37–1.03)	0.59 (0.35–0.99)	116
Max NDVI (1,500)				
Q1 (0.347–0.564)	1.00	1.00	1.00	165
Q2 (0.565–0.608)	0.81 (0.50–1.29)	0.811 (0.50–1.32)	0.78 (0.48–1.28)	121
Q3 (0.609–0.669)	0.56 (0.35–0.88)	0.542 (0.34–0.87)	0.53 (0.33–0.86)	138
Q4 (0.670–0.737)	0.54 (0.31–0.93)	0.53 (0.31–0.93)	0.52 (0.30–0.91)	79
Intermediate-income group (R$ 1,501.00–R$ 2,500.00)				1,060
Max NDVI (200)				
Q1 (0.034–0.217)	1.00	1.00	1.00	288
Q2 (0.218–0.314)	0.67 (0.47–0.96)	0.67 (0.47–0.97)	0.68 (0.47–0.97)	271
Q3 (0.315–0.427)	1.28 (0.91–1.80)	1.26 (0.89–1.79)	1.23 (0.86–1.76)	258
Q4 (0.428–0.699)	0.87 (0.61–1.24)	0.85 (0.59–1.22)	0.88 (0.61–1.28)	243
Max NDVI (400)				
Q1 (0.124–0.361)	1.00	1.00	1.00	290
Q2 (0.362–0.457)	1.13 (0.80–1.59)	1.08 (0.76–1.54)	1.06 (0.75–1.52)	269
Q3 (0.458–0.535)	1.08 (0.77–1.51)	1.083 (0.77–1.53)	1.09 (0.77–1.55)	287
Q4 (0.536–0.714)	0.77 (0.53–1.12)	0.74 (0.51–1.10)	0.77 (0.52–1.14)	213
Max NDVI (1,500)				
Q1 (0.347–0.564)	1.00	1.00	1.00	328
Q2 (0.565–0.608)	0.95 (0.68–1.33)	0.99 (0.70–1.40)	1.03 (0.72–1.46)	261
Q3 (0.609–0.669)	0.912 (0.65–1.30)	0.91 (0.64–1.30)	0.92 (0.65–1.32)	247
Q4 (0.670–0.737)	0.90 (0.63–1.29)	0.88 (0.61–1.27)	0.95 (0.66–1.38)	224
Higher-income group (above R$ 2,500.00)				1,010
Max NDVI (200)				
Q1 (0.034–0.217)	1.00	1.00	1.00	239
Continue				
Q2 (0.218–0.314)	0.75 (0.50–1.12)	0.76 (0.51–1.13)	0.740 (0.49–1.11)	232
Q3 (0.315–0.427)	0.79 (0.54–1.17)	0.80 (0.54–1.17)	0.79 (0.54–1.17)	259
Q4 (0.428–0.699)	0.94 (0.64–1.36)	0.94 (0.64–1.36)	0.98 (0.67–1.44)	280
Max NDVI (400)				
Q1 (0.124–0.361)	1.00	1.00	1.00	210
Q2 (0.362–0.457)	0.73 (0.48–1.10)	0.73 (0.48–1.09)	0.725 (0.48–1.10)	236
Q3 (0.458–0.535)	0.81 (0.55–1.21)	0.83 (0.55–1.24)	0.84 (0.56–1.27)	250
Q4 (0.536–0.714)	0.87 (0.59–1.26)	0.87 (0.59–1.27)	0.92 (0.63–1.35)	314
Max NDVI (1,500)				
Q1 (0.347–0.564)	1.00	1.00	1.00	173
Q2 (0.565–0.608)	0.72 (0.47–1.09)	0.71 (0.47–1.09)	0.71 (0.47–1.10)	241
Q3 (0.609–0.669)	0.77 (0.51–1.17)	0.77 (0.51–1.17)	0.78 (0.52–1.20)	256
Q4 (0.670–0.737)	0.73 (0.49–1.08)	0.74 (0.50–1.10)	0.79 (0.53–1.18)	339

Max NDVI: maximum normalized difference vegetation index; OR: odds ratio.

^a^ Gross OR.

^b^ OR adjusted by sex and age.

^c^ OR adjusted by sex, age, and physical activity.

In the lower-income group (up to BRL 1,500), we observed inverse association in the last quartile of the 400-meter buffer in Model 3, which includes the adjustment by physical activity (Table 2) and in the last two quartiles of the 1,500-meter buffer, both in the bivariate model and in the two models with and without adjustment by physical activity (models 1, 2, and 3). The intermediate-income group presented inverse association in the second quartile of the 200-meter buffer in all models (Table 2), while the higher-income group presented no significant associations. We observed no relevant difference between the models with and without adjustment by physical activity.

## DISCUSSION

This study, conducted with administrative technical staff of a university located in the city of Rio de Janeiro, showed inverse association between the presence of common mental disorders and the exposure to green areas. It was observed that the associations were significant in the lower-income groups and were not significant in the higher-income group.

The results of this study are consistent with other findings presented in the literature. A recent systematic review showed that the scarcity of green areas in the surroundings of the residence is associated with depressive humor^17^ and another review, prior to that, reports evidence of association between the amount of green areas in the surroundings of the residences and better state of mental health^12^. Triguero-Mas et al.^13^, in a study covering the entire region of Catalonia (Spain), found association between the green area surrounding the home, measured by NDVI, and several indicators of mental health, including the presence of CMD. A study in Chicago (USA) found inverse association between NDVI and stress perception, but showed that the extension of the parks had greater association with mental health than the total green area in the neighborhood^18^. Beyer et al.^19^, in a study in Wisconsin (USA), found inverse association between NDVI and symptoms of depression and anxiety.

The associations found in this study varied according to the measure of exposure (mean or maximum NDVI) and the size of the buffers. The maximum NDVI better captured the exposure to green areas among the participants, since the mean NDVI tends to present lower values in urban regions, due to the coexistence of vegetation with uncovered surfaces. In some analyses, associations were observed with maximum NDVI values from 0.218, which reflects the presence of sparse to moderate vegetation in the environment, and in other analyses the associations were observed with higher maximum NDVI values, around 0.609, which corresponds to dense vegetation. As for the buffers, there is no consensus in the literature as to the radius used to obtain the measures of exposure to green areas. In this study, we identified significant associations in buffers of 200, 400, and 1,500 meters, while other studies used buffers with radiuses of 300 and 900 meters and the census sector.

In this study, the associations between green areas and CMD were significant in the low- and intermediate-income groups, while the higher-income group presented no association. This result is possibly a consequence of the difference between these strata. While the low-income population shows greater dependence on the conditions of the neighborhood, as a consequence of the lack of opportunities^11^, the highest-income population has more leisure opportunities and a neighborhood in better general conditions, including exposure to green areas^b^. This finding reinforces the role of income as an effect modifier in the association between green areas and mental disorders. A recent study conducted in England, which also used NDVI as an exposure measure, showed that the effect of green areas on depression was even greater among individuals who resided in residential areas with low socioeconomic status^20^.

The mechanisms by which the green areas act on mental health can be direct and indirect means. The direct means is based on two theories postulated in the field of environmental psychology: the theory of restoration of attention and the theory of psychophysiological reduction of stress^21^. The theory of restoration of attention postulates that the natural environment has the capacity to remedy mental fatigue, stimulating the so-called involuntary attention. Involuntary attention is evoked by interesting or stimulating things in the environment, it is spontaneous and requires no effort. According to this theory, nature is an environment that has a quality called fascination, which at the same time manages to activate involuntary attention and is able to restore direct attention^22^. The theory of psychophysiological reduction of stress emphasizes the emotional affective response that non-threatening natural environments can evoke in human beings, decreasing their surveillance state and consequently stress^23^.

The indirect means postulates that the quality of the physical environment surrounding the residence influences the lifestyle and well-being of the individual^4,24^. Green areas reduce air and noise pollution, the effects of heat islands, make the environment more enjoyable, and encourage a more active and healthy lifestyle, as well as stimulate social contact, which leads to a greater sense of security in the neighborhood^4,24,25^.

In this study, the benefits of urban green areas can occur by the two mechanisms. However, the effect of the practice of physical activities, when adjusted in the analysis, was not very evident. In case part of the effect were due to the practice of physical activities, a reduction of the effect would be expected, that is, that the odds ratio (OR) would approximate the null value. In some cases there was loss of effect, which reinforces the evidence of the mediator role of physical activity; in another case, there was a reduction in the OR value, suggesting an intensification of the protective effect. In any case, the change in these values is very small, in the order of the second decimal place. This result is consistent with those of two previous studies. Triguero-Mas et al.^13^, in Spain, concluded that the surrounding green area was associated with mental health and that physical activity practice and social interaction were probably not the mediators of this association. Richardson et al.^6^, in New Zealand, concluded that greener neighborhoods were associated with mental health and that, although physical activity was more prevalent in these environments, it did not fully explain this relation.

The mechanisms can also vary according to the type of green space. The total vegetation in the housing environment has a direct impact on the reduction of stress, while parks can also mitigate stress by means of social contact^18^. It is important to emphasize that the life habits of individuals are related to the perception of security in the environment. Policies to promote physical activity should consider this aspect^26^, as well as accessibility to leisure areas. A study conducted in Rio de Janeiro showed that violence is a concern related to the use of green areas^27^. Accordingly, it is possible that the indirect mechanism of protection of green areas by their use in the practice of physical activities may still be underutilized in the city.

Vegetation indexes based on remote sensing images have the advantage of covering virtually all regions of the globe. Because they are estimated using uniform methodologies, they facilitate the comparison between different studies, periods, and regions. However, the NDVI is unable to capture information about aspects that influence the use of green areas, such as accessibility, aesthetic quality, security, etc.

Among the limitations of this study, cross-sectional analyses do not estimate the duration of the exposure factor, which in this case refers to the time each individual was exposed to green areas. In the hypothesis that the individual has recently moved to a greener area, it is possible that the beneficial effect on mental health has not yet occurred, as well as the reduction of this effect in case they have recently moved to a region with less vegetation in the surroundings.

It is worth noting that most study participants are people with formal employment, whose time of exposure to green areas around the residence is limited to times before and after work on weekdays and weekends and holidays. The association of the exposure to green areas around the residence is possibly greater in a population that spends more time at home, such as retired people, unemployed people, or people who work at home. Moreover, because we used CMD and not other disorders, such as depression, the associations may have been underestimated, since CMDs do not constitute a diagnosis, being characterized by milder symptoms of anxiety and depression.

Despite the limitations, we observed inverse association between the exposure to green areas in the surroundings of the residence and the presence of common mental disorders. We observed that low-income people benefit more than high-income people and that the association found is not dependent on physical activity. The study advances in the investigation of the role of contextual factors in the distribution of health determinants of the Brazilian population, for which researches of this nature are scarce. Therefore, our findings may help to better understand the impact that urban planning, with preservation or inclusion of green areas, can have on the psychological well-being of people, contributing so this planning seeks greater integration between the different professionals involved in it and in the analysis of health care.

## Footnotes

a Weier J, Herring D. Measuring Vegetation (NDVI & EVI). Greenbelt (MD): NASA Earth Observatory; 2000 [cited 2018 Apr 25]. Available from: https://earthobservatory.nasa.gov/Features/MeasuringVegetation/printall.php

b IBGE. Censo demográfico: 2010 : características urbanísticas do entorno dos domicílios 2010. [cited 2018 Dec 7]. Available from: https://biblioteca.ibge.gov.br/biblioteca-catalogo?id=796&view=detalhes

## References

[1] 1. Sirgy MJ, Cornwell T. How neighborhood features affect quality of life. Soc Indic Res. 2002;59(1):79-114. 10.1023/A:1016021108513

[2] 2. Abdul Ghani S, Nurwati B. Quality of life of residents in urban neighbourhoods of Pulau Pinang, Malaysia. J Constr Dev Ctries. 2012;17(2):117-23.

[3] 3. Fergusson DM, McLeod GFH, Horwood LJ, Swain NR, Chapple S, Poulton R. Life satisfaction and mental health problems (18 to 35 years). Psychol Med. 2015;45(11):2427-36. 10.1017/S0033291715000422 25804325

[4] 4. Lee ACK, Maheswaran R. The health benefits of urban green spaces: a review of the evidence. J Public Health (Oxf). 2011;33(2):212-22. 10.1093/pubmed/fdq068 20833671

[5] 5. Di Nardo F, Saulle R, La Torre G. Green areas and health outcomes: a systematic review of the scientific literature. Ital J Public Health. 2010;7(4):402-13. 10.2427/5699

[6] 6. Richardson EA, Pearce J, Mitchell R, Kingham S. Role of physical activity in the relationship between urban green space and health. Public Health. 2013;127(4):318-24. 10.1016/j.puhe.2013.01.004 23587672

[7] 7. Gladwell VF, Brown DK, Wood C, Sandercock GR, Barton JL. The great outdoors: how a green exercise environment can benefit all. Extrem Physiol Med. 2013;2(1):3. 10.1186/2046-7648-2-3 PMC3710158 23849478

[8] 8. Gonçalves DA, Mari JJ, Bower P, Gask L, Dowrick C, Tófoli LF, et al. Brazilian multicentre study of common mental disorders in primary care: rates and related social and demographic factors. Cad Saude Publica. 2014;30(3):623-32. 10.1590/0102-311X00158412 24714951

[9] 9. Moraes RSM, Silva DAS, Oliveira WF, Peres MA. Social inequalities in the prevalence of common mental disorders in adults: a population-based study in Southern Brazil. Rev Bras Epidemiol. 2017;20(1):43-56. 10.1590/1980-5497201700010004 28513793

[10] 10. Fonseca MLG, Guimarães MBL, Vasconcelos EM. Sofrimento difuso e transtornos mentais comuns: uma revisão bibliográfica. Rev APS. 2008;11(3):285-94.

[11] 11. Vries S, Verheij RA, Groenewegen PP, Spreeuwenberg P. Natural environments - healthy environments? An exploratory analysis of the relationship between greenspace and health. Environ Plan A. 2003;35(10):1717-31. 10.1068/a35111

[12] 12. Gascon M, Triguero-Mas M, Martínez D, Dadvand P, Forns J, Plasència A, et al. Mental health benefits of long-term exposure to residential green and blue spaces: a systematic review. Int J Environ Res Public Health. 2015;12(4):4354-79. 10.3390/ijerph120404354 PMC4410252 25913182

[13] 13. Triguero-Mas M, Dadvand P, Cirach M, Martínez D, Medina A, Mompart A, et al. Natural outdoor environments and mental and physical health: relationships and mechanisms. Environ Int. 2015;77:35-41. 10.1016/j.envint.2015.01.012 25638643

[14] 14. Silveira IH, Junger WL. Green spaces and mortality due to cardiovascular diseases in the city of Rio de Janeiro. Rev Saude Publica. 2018;52:49. 10.11606/S1518-8787.2018052000290 PMC5947462 29723390

[15] 15. Faerstein E, Chor D, Lopes CS, Werneck GL. Estudo Pró-Saúde: características gerais e aspectos metodológicos. Rev Bras Epidemiol. 2005;8(4):454-66. 10.1590/S1415-790X2005000400014

[16] 16. Goldberg D, Williams P. A user’s guide to the general health questionnaire. Windsor: NFER-Nelson; 1988

[17] 17. Rautio N, Filatova S, Lehtiniemi H, Miettunen J. Living environment and its relationship to depressive mood: a systematic review. Int J Soc Psychiatry. 2018;64(1):92-103. 10.1177/0020764017744582 29212385

[18] 18. Fan Y, Das KV, Chen Q. Neighborhood green, social support, physical activity, and stress: assessing the cumulative impact. Health Place. 2011;17(6):1202-11. 10.1016/j.healthplace.2011.08.008 21920795

[19] 19. Beyer KMM, Kaltenbach A, Szabo A, Bogar S, Javier Nieto F, Malecki KM. Exposure to neighborhood green space and mental health: evidence from the survey of the health of Wisconsin. Int J Environ Res Public Health. 2014;11(3):3453-72. 10.3390/ijerph110303453 PMC3987044 24662966

[20] 20. Sarkar C, Webster C, Gallacher J. Residential greenness and prevalence of major depressive disorders: a cross-sectional, observational, associational study of 94 879 adult UK Biobank participants. Lancet Planet Health. 2018;2(4):e162-73. 10.1016/S2542-5196(18)30051-2 29615217

[21] 21. Gressler SC, Günther, IA. Ambientes restauradores: definição, histórico, abordagens e pesquisas. Estud Psicol (Natal). 2013;18(3):487-95. 10.1590/S1413-294X2013000300009

[22] 22. Kaplan S. The restorative benefits of nature: toward an integrative framework. J Environ Psychol. 1995;15(3):169-82. 10.1016/0272-4944(95)90001-2

[23] 23. Ulrich RS. Aesthetic and affective response to natural environment. In: Altman I, Wohwill, editors. Behavior and the natural environment. Boston, MA: Springer US; 1983. p. 85-125.

[24] 24. Tzoulas K, Korpela K, Venn S, Yli-Pelkonen V, Kaźmierczak A, Niemela J, et al. Promoting ecosystem and human health in urban areas using Green Infrastructure: a literature review. Landsc Urban Plan. 2007;81(3):167-78. 10.1016/j.landurbplan.2007.02.001

[25] 25. Putrik P, Vries NK, Mujakovic S, Amelsvoort L, Kant I, Kunst AE, et al. Living environment matters: relationships between neighborhood characteristics and health of the residents in a Dutch municipality. J Community Health. 2015;40(1):47-56. 10.1007/s10900-014-9894-y 24917124

[26] 26. Florindo AA, Salvador EP, Reis RS. Physical activity and its relationship with perceived environment among adults living in a region of low socioeconomic level. J Phys Act Health. 2013;10(4):563-71. 10.1123/jpah.10.4.563 22976232

[27] 27. Costa MAM. Uso e percepção de áreas verdes em grandes cidades: estudo de caso em dois parques urbanos no Rio de Janeiro [dissertação]. Rio de Janeiro: Pontifícia Universidade Católica do Rio de Janeiro; 2016.

