# Supplementary material for: Is living near green areas beneficial to mental health? Results of the Pró-Saúde Study
Source: Rev Saude Publica. 2019 Sep 12;53:75. doi: 10.11606/s1518-8787.2019053001008 (PMC6752646; doi:10.11606/s1518-8787.2019053001008)
Supplement: Translation

# Translation: Morar perto de áreas verdes é benéfico para a saúde mental? Resultados do Estudo Pró-Saúde

**Authors:** Patricia Amado Barreto, Claudia Souza Lopes, Ismael Henrique da Silveira, Eduardo Faerstein, Washington Leite Junger

**Affiliations:** I Universidade do Estado do Rio de Janeiro. Instituto de Medicina Social. Programa de Pós-Graduação em Saúde Coletiva. Rio de Janeiro, RJ, Brasil; II Universidade do Estado do Rio de Janeiro. Instituto de Medicina Social. Departamento de Epidemiologia. Rio de Janeiro, RJ, Brasil

**Keywords:** Adulto, Transtornos Mentais, prevenção & controle, Áreas Verdes, Fatores Socioeconômicos, Saúde Mental, Estudos Transversais

## Abstract

**OBJETIVO:**

Investigar a associação entre a exposição às áreas verdes no entorno da residência e a presença de transtornos mentais comuns entre adultos, segundo diferentes estratos de renda.

**MÉTODOS:**

Estudo seccional com 2.584 participantes do Estudo Pró-Saúde (2006), residentes na cidade do Rio de Janeiro. Os transtornos mentais comuns foram aferidos por meio do *General Health Questionnaire (GHQ-12)* e a exposição às áreas verdes pelo índice de vegetação por diferença normalizada, em *buffers* com raios entre 100 e 1.500 metros em torno da residência. Foram utilizados o índice de vegetação por diferença normalizada médio e máximo categorizado em quartis. A população do estudo foi dividida em três subgrupos, de acordo com a renda: baixa, intermediária e alta. Foram estimadas razões de chances e seus intervalos de 95% de confiança com modelos de regressão logística. Os modelos foram ajustados por sexo e idade, com e sem inclusão da prática de atividade física.

**RESULTADOS:**

A proporção de transtornos mentais comuns foi de 30% e 39% entre homens e mulheres, respectivamente. Os resultados dos modelos ajustados mostraram associação inversa entre a presença de áreas verdes no entorno do domicílio e a ocorrência de transtornos mentais comuns, no *buffer* de 200 metros no grupo de renda intermediária e nos *buffers* de 400 e 1.500 metros no grupo de baixa renda. A razão de chances variou de 0,52 (*buffer* de 1.500 metros) a 0,68 (*buffer* de 200 metros). A associação encontrada foi independente da prática de atividade física.

**CONCLUSÕES:**

As evidências encontradas sugerem a existência de um efeito benéfico de áreas verdes urbanas na saúde mental dos indivíduos de renda mais baixa. Tais achados podem ajudar na compreensão de como o meio ambiente urbano pode afetar a saúde mental da população.

## INTRODUÇÃO

O ambiente físico da vizinhança, incluindo aspectos como paisagem do bairro, aglomeração e nível de ruído, estabelecimentos escolares e de saúde, instalações públicas recreativas e transporte público, contribui para a qualidade de vida e o sentimento de satisfação dos residentes em relação ao bairro, à moradia e à comunidade^1,2^. A satisfação com a vida apresenta relação com os transtornos mentais^3^ e as características físicas do ambiente da vizinhança podem ter o potencial de influenciar a saúde mental por meio da melhoria da qualidade de vida dos seus residentes. Estudos demonstram que a disponibilidade de áreas verdes (praças, parques, jardins, bosques e ruas arborizadas) tem o potencial de provocar a sensação de relaxamento, bem-estar e coesão social^4,5^, além de promover a prática de atividades físicas^6,7^, o que contribui para a promoção da saúde mental na população.

Estudos têm mostrado uma elevada prevalência de transtornos mentais comuns (TMC) em áreas urbanas no país^8,9^. Tais transtornos são caracterizados pela presença de sintomas de ansiedade e depressão que, apesar de não serem suficientes para a caracterização de um diagnóstico psiquiátrico, podem causar sofrimento substancial e prejuízo na vida dos indivíduos^10^.

Existem evidências de que pessoas com status socioeconômico mais baixo são mais dependentes do entorno e apresentam piores condições de saúde de uma maneira geral^11^. Medidas que visem promover a melhora das características físicas nos centros urbanos poderiam impactar a saúde mental da parcela mais carente da população brasileira, ajudando a diminuir a desigualdade em relação à qualidade de vida.

A maioria dos estudos sobre este tema se concentra em países de alta renda, e a associação entre exposição às áreas verdes e saúde mental é ainda inconsistente, havendo escassas evidências sobre o tema em países de renda baixa e média, como o Brasil e outros países da América Latina^4,12^. A mediação dos potenciais efeitos benéficos pela via do favorecimento de práticas de atividade física ainda não apresenta resultados conclusivos^6,13^. O Rio de Janeiro, segunda maior cidade do país, possui um ambiente natural que, apesar de exuberante, é desigualmente distribuído pela cidade^14^. Dado esse cenário, o objetivo deste estudo foi investigar a associação entre a presença de áreas verdes no entorno da residência e a ocorrência de TMC entre adultos residentes e entre diferentes estratos econômicos.

## MÉTODOS

### Desenho e População de Estudo

Trata-se de um estudo transversal inserido em um estudo longitudinal, o Estudo Pró-Saúde (EPS). O objetivo do EPS é investigar o papel de determinantes de saúde e morbidade entre funcionários técnicos administrativos de uma universidade localizada na cidade do Rio de Janeiro, Brasil^15^.

Para o presente estudo, foram utilizados dados da terceira fase do EPS, realizada no ano de 2006. Dentre os 3.604 participantes desta fase, foram incluídos no estudo apenas aqueles que residiam no município do Rio de Janeiro e cujos endereços foram georreferenciados com sucesso, perfazendo 2.584 indivíduos.

### Variáveis Individuais

A coleta de dados foi realizada por meio de questionário autopreenchível, multidimensional, abrangendo morbidade física e mental, acesso e utilização dos serviços de saúde e aspectos socioeconômicos, entre outros. Foram utilizados procedimentos visando a qualidade da informação, como estudo-piloto, teste-reteste para assegurar a confiabilidade do instrumento e procedimentos de dupla digitação independente^15^.

O desfecho analisado neste estudo foi a ocorrência de transtorno mental comum, medida pela versão de 12 itens do *General Health Questionnaire* (GHQ-12), um instrumento de rastreamento de transtornos mentais não psicóticos, incluindo sintomas de ansiedade, depressão e somáticos. Cada pergunta tem quatro opções de resposta, sendo duas consideradas positivas e duas negativas que, respectivamente, somam 1 ou 0 ao escore do GHQ. O participante é classificado como positivo para TMC se o escore for maior ou igual a 3^16^.

Outras variáveis individuais consideradas no estudo foram: sexo, idade em anos (de 25 a 35, de 36 a 45, de 46 a 55, de 56 a 65, de 66 a 75), renda mensal líquida (até 500 reais, 501 a 1.000 reais, 1.001 a 1.500 reais, 1.501 a 2.000 reais, 2.001 a 2.500 reais, 2.501 a 3.000 reais, 3.001 a 4.000 reais, 4.001 a 5.000 reais, acima de 5.000) e prática de atividades físicas (sim ou não). Esta foi avaliada por meio da pergunta: “Nas últimas duas semanas, você praticou alguma atividade física para melhorar sua saúde, condição física ou com objetivo estético ou de lazer?”.

### Indicadores de Exposição às Áreas Verdes

Para obter as variáveis de exposição no entorno das residências, os endereços dos participantes foram georreferenciados por meio da função de georreferenciamento (*Google Geocoding API*) da interface de programação de aplicativos (*application programming interface* – API) do Google. A API relaciona os endereços informados com a base de dados do Google Maps para a captura das coordenadas latitude e longitude. O procedimento foi realizado no programa R 3.1.1.

As áreas verdes, neste trabalho, se referem a qualquer vegetação no entorno da residência, desde árvores plantadas nas calçadas, canteiros ou praças, jardins públicos ou residenciais, até reservas, parques ou áreas de recreação, desde que cobertas por algum tipo de vegetação. A exposição foi estimada pelo índice de vegetação por diferença normalizada (*normalized difference vegetation index* – NDVI).

O NDVI é calculado a partir de imagens de sensoriamento remoto por satélite e mede a densidade da vegetação de uma região. A vegetação produz um padrão de baixa refletância do comprimento de onda vermelho e alta refletância próximo do infravermelho. O índice é determinado pela diferença das duas bandas dividida pela soma. Cada pixel da imagem recebe um valor do NDVI que pode variar entre 1 e -1, assumindo na prática valores entre -0,1 e 0,7. Valores negativos indicam a presença de água, gelo e nuvens; entre -0,1 e 0,1, superfícies descobertas; e em torno de 0,6, a vegetação verde densa^a^.

As imagens foram obtidas da coleção LandSat 4-5 TM, do Serviço Geológico dos Estados Unidos (*United States Geological Survey* – USGS), que tem precisão de 30 metros. Para o ano de 2006 e para a região do estudo, estavam disponíveis três imagens que apresentavam menos de 10% de nuvens, correspondentes aos meses de fevereiro, agosto e dezembro. O NDVI do período de estudo foi estimado pela média destes três meses. A exposição dos participantes foi mensurada pelo NDVI máximo e médio em *buffers,* regiões circulares centradas nas residências dos participantes, com raios de 100, 200, 300, 400, 500, 1.000 e 1.500 metros. Foram utilizados *buffers* de diferentes tamanhos para realizar análises de sensibilidade, já que não há consenso na literatura sobre o tamanho adequado. O NDVI médio é calculado pela soma do NDVI de cada pixel dividido pelo número de pixels dentro do *buffer*. O NDVI máximo se refere ao valor mais elevado do NDVI em um pixel dentro do *buffer*.

### Análise dos Dados

Os participantes foram estratificados em três grupos de renda (renda baixa: até 1.500 reais, renda intermediária: de 1.501 a 2.500 reais, renda alta: acima de 2.501 reais). Foram testadas as associações entre a exposição às áreas verdes e a presença de TMC por meio de modelos de regressão logística bivariados e multivariados nesses três grupos. Ainda foram analisados dois modelos multivariados, um sem ajustar pela prática de atividades físicas e outro ajustado por essa variável, para investigação de sua influência no modelo. A correlação e a potencial colinearidade entre as variáveis explicativas foi testada pelo coeficiente de correlação de Spearman e pelo fator de inflação de variância (*variance inflation factor* – VIF). As análises foram realizadas no programa R 3.1.1.

A pesquisa foi aprovada inicialmente na linha de base do estudo longitudinal em 1999 pelo Comitê de Ética e Pesquisa do Hospital Pedro Ernesto e posteriormente pelo Comitê de Ética em Pesquisa do Instituto de Medicina Social da Universidade do Estado do Rio de Janeiro (Registro CAAE 0041.0.259.000-11). Todos os participantes assinaram termo de consentimento livre e esclarecido.

## RESULTADOS

A Tabela 1 descreve a presença de TMC segundo variáveis individuais na população de estudo. Os TMC foram mais frequentes entre mulheres, participantes das faixas etárias de 25 a 35 anos e de 66 a 75 anos, participantes com renda mais baixa e os que não praticaram atividade física nas últimas duas semanas.

**Tabela 1:** Ocorrência de TMC, segundo variáveis individuais nos participantes do Estudo Pró-Saúde, residentes no município do Rio de Janeiro, Brasil, 2006.

Variável	Participantes		Ocorrência de TMC	
	
	n	%	%	p*
Sexo				
Masculino	1.062	41,10	30,23	< 0,01
Feminino	1.522	58,90	39,36	
Faixa etária (anos)				
25–35	184	7,12	44,02	0,02
36–45	936	36,22	34,83	
46–55	1.094	42,34	36,20	
56–65	319	12,35	29,78	
66–75	50	1,93	42,00	
Renda líquida (reais)				
≤ 500	52	2,01	57,69	< 0,01
501–1.000	150	5,80	42,67	
1.001–1.500	301	11,65	49,17	
1.501–2.000	373	14,43	36,73	
2.001–2.500	321	12,42	36,14	
2.501–3.000	366	14,16	34,97	
3.001–4.000	345	13,35	31,01	
4.001–5.000	249	9,64	30,12	
> 5.000	416	16,10	26,20	
Prática de atividade física				
Não	1.453	56,23	42,60	< 0,01
Sim	1.128	43,65	26,60	

*Teste qui-quadrado

A média calculada para o NDVI médio foi maior conforme o tamanho do *buffer*, mas sempre apresentando valores baixos. O menor valor foi encontrado no *buffer* de 100 metros (0,099) e o maior, no *buffer* de 1.500 metros (0,154). A média do NDVI máximo também aumentou conforme o tamanho do *buffer*; o menor valor foi de 0,233 no *buffer* de 100 metros e o maior valor, de 0,611 no *buffer* de 1.500 metros.

A Tabela 2 apresenta os resultados das análises brutas e ajustadas da associação entre a presença de TMC e a exposição às áreas verdes medida pelo NDVI máximo, estratificada pela renda. O modelo 1 apresenta as análises brutas, o modelo 2 apresenta as análises ajustadas por sexo e idade e o modelo 3 apresenta o ajuste pelas variáveis individuais do modelo 2 mais a prática de atividade física. Estão exibidos nessa tabela apenas os resultados dos *buffers* que apresentaram associação estatisticamente significativa, ao nível de 5%.

**Tabela 2:** Associações entre a ocorrência de transtornos mentais comuns (TMC) e a exposição às áreas verdes, medida pelo NDVI máximo em *buffers* de diferentes tamanhos, estratificadas por renda. Estudo Pró-Saúde, Rio de Janeiro, Brasil, 2006.

NDVI nos *buffers* (metros)	Modelo 1^a^	Modelo 2^b^	Modelo 3^c^	n
Grupo de renda baixa (até R$ 1.500,00)				503
NDVI máx. (200)				
Q1 (0,034–0,217)	1,00	1,00	1,00	114
Q2 (0,218–0,314)	0,87 (0,53–1,42)	0,88 (0,53–1,46)	0,88 (0,53–1,48)	142
Q3 (0,315–0,427)	1,07 (0,64–1,77)	1,071 (0,637–1,80)	1,02 (0,60–1,72)	126
Q4 (0,428–0,699)	0,82 (0,49–1,37)	0,74 (0,43–1,26)	0,72 (0,42–1,24)	120
NDVI máx. (400)				
Q1 (0,124–0,361)	1,00	1,00	1,00	144
Q2 (0,362–0,457)	0,76 (0,48–1,22)	0,81 (0,50–1,31)	0,78 (0,48–1,27)	137
Q3 (0,458–0,535)	0,97 (0,57–1,57)	1,01 (0,60–1,70)	0,99 (0,58–1,67)	105
Q4 (0,536–0,714)	0,63 (0,39–1,04)	0,62 (0,37–1,03)	0,59 (0,35–0,99)	116
NDVI máx. (1.500)				
Q1 (0,347–0,564)	1,00	1,00	1,00	165
Q2 (0,565–0,608)	0,81 (0,50–1,29)	0,811 (0,50–1,32)	0,78 (0,48–1,28)	121
Q3 (0,609–0,669)	0,56 (0,35–0,88)	0,542 (0,34–0,87)	0,53 (0,33–0,86)	138
Q4 (0,670–0,737)	0,54 (0,31–0,93)	0,53 (0,31–0,93)	0,52 (0,30–0,91)	79
Grupo de renda intermediária (R$ 1.501,00–R$ 2.500,00)				1.060
NDVI máx. (200)				
Q1 (0,034–0,217)	1,00	1,00	1,00	288
Q2 (0,218–0,314)	0,67 (0,47–0,96)	0,67 (0,47–0,97)	0,68 (0,47–0,97)	271
Q3 (0,315–0,427)	1,28 (0,91–1,80)	1,26 (0,89–1,79)	1,23 (0,86–1,76)	258
Q4 (0,428–0,699)	0,87 (0,61–1,24)	0,85 (0,59–1,22)	0,88 (0,61–1,28)	243
NDVI máx. (400)				
Q1 (0,124–0,361)	1,00	1,00	1,00	290
Q2 (0,362–0,457)	1,13 (0,80–1,59)	1,08 (0,76–1,54)	1,06 (0,75–1,52)	269
Q3 (0,458–0,535)	1,08 (0,77–1,51)	1,083 (0,77–1,53)	1,09 (0,77–1,55)	287
Q4 (0,536–0,714)	0,77 (0,53–1,12)	0,74 (0,51–1,10)	0,77 (0,52–1,14)	213
NDVI máx. (1.500)				
Q1 (0,347–0,564)	1,00	1,00	1,00	328
Q2 (0,565–0,608)	0,95 (0,68–1,33)	0,99 (0,70–1,40)	1,03 (0,72–1,46)	261
Q3 (0,609–0,669)	0,912 (0,65–1,30)	0,91 (0,64–1,30)	0,92 (0,65–1,32)	247
Q4 (0,670–0,737)	0,90 (0,63–1,29)	0,88 (0,61–1,27)	0,95 (0,66–1,38)	224
Grupo de renda mais elevada (acima de R$ 2.500,00)				1.010
NDVI máx. (200)				
Q1 (0,034–0,217)	1,00	1,00	1,00	239
Q2 (0,218–0,314)	0,75 (0,50–1,12)	0,76 (0,51–1,13)	0,740 (0,49–1,11)	232
Q3 (0,315–0,427)	0,79 (0,54–1,17)	0,80 (0,54–1,17)	0,79 (0,54–1,17)	259
Q4 (0,428–0,699)	0,94 (0,64–1,36)	0,94 (0,64–1,36)	0,98 (0,67–1,44)	280
NDVI máx. (400)				
Q1 (0,124–0,361)	1,00	1,00	1,00	210
Q2 (0,362–0,457)	0,73 (0,48–1,10)	0,73 (0,48–1,09)	0,725 (0,48–1,10)	236
Q3 (0,458–0,535)	0,81 (0,55–1,21)	0,83 (0,55–1,24)	0,84 (0,56–1,27)	250
Q4 (0,536–0,714)	0,87 (0,59–1,26)	0,87 (0,59–1,27)	0,92 (0,63–1,35)	314
NDVI máx. (1.500)				
Q1 (0,347–0,564)	1,00	1,00	1,00	173
Q2 (0,565–0,608)	0,72 (0,47–1,09)	0,71 (0,47–1,09)	0,71 (0,47–1,10)	241
Q3 (0,609–0,669)	0,77 (0,51–1,17)	0,77 (0,51–1,17)	0,78 (0,52–1,20)	256
Q4 (0,670–0,737)	0,73 (0,49–1,08)	0,74 (0,50–1,10)	0,79 (0,53–1,18)	339

NDVI máx.: índice de vegetação por diferença normalizada máximo; RC: razão de chances.

^a^ RC brutas.

^b^ RC ajustadas por sexo e idade.

^c^ RC ajustadas por sexo, idade e atividade física.

No grupo de renda mais baixa (até 1.500 reais), observou-se associação inversa no último quartil do *buffer* de 400 metros no modelo 3, que inclui o ajuste por atividade física (Tabela 2) e nos dois últimos quartis do *buffer* de 1.500 metros, tanto no modelo bivariado quanto nos dois modelos sem e com ajuste por atividade física (modelos 1, 2 e 3). O grupo de renda intermediária apresentou associação inversa no segundo quartil do *buffer* de 200 metros em todos os modelos (Tabela 2), enquanto o grupo de renda mais alta não apresentou associações significativas. Não foi observada diferença relevante entre os modelos com e sem ajuste por atividade física.

## DISCUSSÃO

O presente estudo, realizado com funcionários técnicos administrativos de uma universidade localizada na cidade do Rio de Janeiro, mostrou associação inversa entre a presença de transtornos mentais comuns e a exposição a áreas verdes. Observou-se que as associações foram significativas nos grupos de rendas mais baixas e não foram significativas no grupo de renda mais alta.

Os resultados deste estudo vão ao encontro de outras evidências apresentadas na literatura. Uma revisão sistemática recente mostrou que a escassez de verde no entorno da residência está associada com humor depressivo^17^ e outra, anterior, relata evidências de associação entre a quantidade de verde no entorno das residências e melhor estado de saúde mental^12^. Triguero-Mas et al.^13^, em um estudo abrangendo toda a região da Catalunha (Espanha), encontraram associação entre o verde no entorno do domicílio, medido pelo NDVI, e vários indicadores de saúde mental, incluindo a presença de TMC. Um estudo em Chicago (EUA) encontrou associação inversa entre o NDVI e a percepção do estresse, porém evidenciou que a extensão dos parques apresentava maior associação com a saúde mental do que o total de verde na vizinhança^18^. Beyer et al.^19^, em um estudo em Wisconsin (EUA), encontraram associação inversa entre o NDVI e sintomas de depressão e ansiedade.

As associações encontradas neste estudo variaram conforme a medida de exposição (NDVI médio ou máximo) e o tamanho dos *buffers*. O NDVI máximo capturou melhor a exposição ao verde entre os participantes, pois o NDVI médio tende a apresentar valores mais baixos em regiões urbanas, devido à coexistência de vegetação com superfícies descobertas Em algumas análises foram observadas associações com valores de NDVI máximo a partir de 0,218, que reflete a presença de vegetação esparsa a moderada no ambiente, e em outras análises as associações foram observadas com valores de NDVI máximo mais elevados, em torno de 0,609, que corresponde à vegetação densa. Quanto aos *buffers,* não há um consenso na literatura a respeito do raio utilizado para obter as medidas de exposição ao verde. No presente estudo, foram identificadas associações significativas em *buffers* de 200, 400 e 1.500 metros, enquanto outros estudos utilizaram *buffers* com raio de 300^13^ e 900 metros^18^ e o setor censitário^19^.

Neste estudo, as associações entre áreas verdes e TMC foram significativas nos grupos de renda baixa e intermediária, enquanto o grupo de maior renda não as apresentou. É possível que esse resultado seja consequência da diferença entre esses estratos. Enquanto a população de baixa renda apresenta uma dependência maior das condições da vizinhança, em consequência da carência de oportunidades^11^, a população de renda mais alta tem mais oportunidades de lazer e uma vizinhança em melhores condições gerais, incluindo exposição ao verde^b^. Tal achado reforça o papel da renda como modificadora de efeito na associação entre áreas verdes e transtornos mentais. Estudo recente conduzido na Inglaterra, que também utilizou o NDVI como medida de exposição, mostrou que o efeito das áreas verdes na depressão era ainda maior entre indivíduos que residiam em áreas residenciais de baixo status socioeconômico^20^.

Os mecanismos pelos quais as áreas verdes atuam na saúde mental podem se dar por via direta e indireta. A via direta se baseia em duas teorias postuladas no campo da psicologia ambiental: a teoria da restauração da atenção e a teoria da redução psicofisiológica do estresse^21^. A teoria da restauração da atenção postula que o ambiente natural tem a capacidade de restaurar a fadiga mental, estimulando a chamada atenção involuntária. A atenção involuntária é evocada por coisas interessantes ou estimulantes no ambiente, é espontânea e não demanda esforço. Segundo essa teoria, a natureza é um ambiente que possui uma qualidade denominada fascinação, que, ao mesmo tempo que consegue ativar a atenção involuntária, é capaz de restaurar a atenção direta^22^. A teoria da redução psicofisiológica do estresse enfatiza a resposta afetiva emocional que ambientes naturais não ameaçadores podem evocar no ser humano, diminuindo seu estado de vigilância e consequentemente o estresse^23^.

A via indireta postula que a qualidade do ambiente físico no entorno da residência influencia o estilo de vida e o bem-estar do indivíduo^4,24^. Áreas verdes reduzem a poluição do ar e sonora, os efeitos das ilhas de calor, tornam o ambiente mais agradável e incentivam um estilo de vida mais ativo e saudável, além de estimular o contato social, o que tem como consequência maior sensação de segurança na vizinhança^4,24,25^.

No presente estudo, os benefícios das áreas verdes urbanas podem se dar pelos dois mecanismos. No entanto, o efeito da prática de atividades físicas, quando ajustado na análise, não foi muito evidente. Caso parte do efeito se desse pela prática de atividades físicas, se esperaria uma redução do efeito, ou seja, que a razão de chances (RC) se aproximasse do valor nulo. Em alguns casos houve perda de efeito, o que reforça a evidência do papel mediador da atividade física; em outro, houve redução do valor da RC, sugerindo uma acentuação do efeito protetor. De todo modo, a alteração desses valores é muito pequena, na ordem da segunda casa decimal. Esse resultado é consistente com os de dois estudos anteriores. Triguero-Mas et al.^13^, na Espanha, concluíram que o verde do entorno estava associado à saúde mental e que a prática de atividade física e interação social provavelmente não eram os mediadores desta associação. Richardson et al.^6^, na Nova Zelândia, concluíram que vizinhanças mais verdes estavam associadas à saúde mental e que, embora a atividade física fosse maior nestes ambientes, ela não explicava totalmente essa relação.

Os mecanismos ainda podem variar conforme o tipo de espaço verde. A vegetação total no ambiente de moradia tem um impacto direto na redução do estresse, enquanto os parques podem também atenuar o estresse por meio do contato social^18^. É importante ressaltar que os hábitos de vida dos indivíduos estão relacionados com a percepção da segurança no ambiente. Políticas de promoção da atividade física devem levar em conta esse aspecto^26^, assim como a acessibilidade às áreas de lazer. Um estudo realizado no Rio de Janeiro mostrou que a violência é uma preocupação relacionada ao uso das áreas verdes^27^. Nesse sentido, é possível que o mecanismo indireto de proteção do verde por sua utilização na prática de atividades físicas possa ainda estar subaproveitado na cidade.

Os índices de vegetação baseados em imagens de sensoriamento remoto têm a vantagem de cobrir praticamente todas as regiões do globo. Por serem calculados utilizando metodologias uniformes, facilitam a comparação entre diferentes estudos, períodos e regiões. Entretanto, o NDVI não consegue captar informações acerca de aspectos que influenciam a utilização das áreas verdes, como acessibilidade, qualidade estética, segurança etc.

Dentre as limitações deste estudo, as análises transversais não estimam por quanto tempo atuou o fator de exposição, que neste caso se refere ao tempo de exposição de cada indivíduo às áreas verdes. Na hipótese de o indivíduo ter se mudado recentemente para uma área mais verde, é possível que o efeito benéfico para a saúde mental ainda não tenha acontecido, assim como a redução desse efeito se ele tiver se mudado recentemente para uma região com menos vegetação no entorno.

Cabe ressaltar que a maioria dos participantes do estudo é composta por pessoas com emprego formal, cujo tempo de exposição às áreas verdes no entorno do domicílio é limitado a momentos antes e depois do trabalho nos dias úteis e aos finais de semana e feriados. É possível que a associação da exposição às áreas verdes no entorno do domicilio seja maior em uma população que passe mais tempo em casa, como aposentados, desempregados ou pessoas que trabalham em casa. Além disso, o fato de termos utilizado TMC e não outros transtornos, como a depressão, pode ter subestimado as associações, já que os TMC não constituem um diagnóstico, sendo caracterizados por sintomas mais leves de ansiedade e depressão.

Apesar das limitações, foi possível observar a associação inversa entre a exposição às áreas verdes no entorno do domicílio e a presença de transtornos mentais comuns. Observou-se que as pessoas de baixa renda se beneficiam mais do que as pessoas de alta renda e que a associação encontrada não é dependente da atividade física. O estudo avança na investigação do papel de fatores contextuais na distribuição de determinantes de saúde da população brasileira, para a qual são raras as pesquisas dessa natureza. Nossos achados podem, portanto, auxiliar na melhor compreensão do impacto que o planejamento urbano, com a preservação ou a inclusão de áreas verdes, pode ter no bem-estar psicológico das pessoas, fornecendo subsídios para que tal planejamento busque uma maior integração entre os diferentes profissionais envolvidos nele e na análise dos cuidados de saúde.

## Footnotes

a Weier J, Herring D. Measuring Vegetation (NDVI & EVI). Greenbelt (MD): NASA Earth Observatory; 2000 [citado 25 abr 2018]. Disponível em: https://earthobservatory.nasa.gov/Features/MeasuringVegetation/printall.php

b IBGE. Censo demográfico : 2010 : características urbanísticas do entorno dos domicílios 2010. [citado 7 dez 2018] Disponível em: https://biblioteca.ibge.gov.br/biblioteca-catalogo?id=796&view=detalhes
